# Poly(methacrylic Acid)-Cellulose Brushes as Anticancer Porphyrazine Carrier

**DOI:** 10.3390/nano11081997

**Published:** 2021-08-03

**Authors:** Elena L. Krasnopeeva, Elena Yu. Melenevskaya, Larisa G. Klapshina, Natalia Yu. Shilyagina, Irina V. Balalaeva, Nikolay N. Smirnov, Michael A. Smirnov, Alexander V. Yakimansky

**Affiliations:** 1 Institute of Macromolecular Compounds, Russian Academy of Sciences, 199004 Saint Petersburg, Russia; opeeva@gmail.com (E.L.K.); meleneyu@yandex.ru (E.Y.M.); rambow@inbox.ru (N.N.S.); smirnov_michael@mail.ru (M.A.S.); 2 Razuvaev Institute of Organomettalic Chemistry, Russian Academy of Sciences, 603137 Nizhniy Novgorod, Russia; klarisa@iomc.ras.ru; 3 Institute of Biology and Biomedicine, Nizhniy Novgorod State University, 603950 Nizhniy Novgorod, Russia; nat-lekanova@yandex.ru (N.Y.S.); irin-b@mail.ru (I.V.B.); 4 Institute of Chemistry, Saint Petersburg State University, 198504 Saint Petersburg, Russia

**Keywords:** cellulose brush, ATRP, PDT, drug carrier, porphyrazine

## Abstract

The prospective strategy for treatment of cancer is based on the application of nano-sized macromolecular carriers, which are able penetrate inside and can be accumulated within tumor tissue. In this work graft copolymers of cellulose and poly(methacrylic acid) has been prepared and tested as a nanocontainers for the delivery of drug to tumor. For this purpose, two derivatives of porphyrazine suitable for photodynamic cancer therapy were loaded into prepared polymer brush. Fluorescence imaging was applied for monitoring of accumulation of porphyrazine in the CT26 murine colon carcinoma. The selective accumulation of cellulose brush loaded with porphyrazine in tumor was demonstrated by fluorescence intensity contrast between the tumor area and normal tissues. The tumor growth rate after photodynamic therapy were assessed and inhibition of its growth was revealed.

## 1. Introduction

One prospective strategy for treatment of cancer is based on the application of nano-sized macromolecular carriers, which are able to penetrate inside, and can be accumulated within, tumor tissue [1,2,3,4]. This accumulation mechanism is often referred to as enhanced permeability and retention (EPR) [5,6,7,8]. The reason for the possibility of such phenomenon is the immaturity of the membrane of the endothelial cells and the anomalous basement membrane of capillaries in the tumor tissue, as well as the overproduction of proinflammatory mediators of the vessels that contribute to increased vascular permeability. These problems are widely discussed in today’s numerous reviews [9,10,11,12,13,14,15]. In the tumor tissue, intercellular gap can be in the range 20–2000 nm [16], while in the vasculature in health tissue it is not higher than 5 nm. At the same time, the deletion of particles, which penetrate to the tumor tissue through the blood vessels is hampered because of disorders in the lymphatic vessels in the tumor tissue. Thus, in order to improve the therapeutic benefits of nanomedicines, particular attention is paid to the development of carriers with appropriate size in combination with sorption/release properties toward the low molecular weight drugs. Liposomes are some of the most investigated drug-carriers because they are biocompatible, non-immunogenic, demonstrate possibility for self-assembly and the ability to absorb hydrophilic or hydrophobic drugs resulting in an improvement of their solubility and protection of the encapsulated molecules from the external media [17,18,19,20]. However, the disadvantages of liposomes, such as low solubility and short half-life are also mentioned [17]. At the same time, organic and inorganic particles demonstrate some distinctive features, which makes them more attractive than liposomes [21,22]. Application of synthetic organic particles makes it possible to create carriers with beneficial properties by rational molecular design, which is provided by using of modern synthetic methods, that allow tuning of the chemical functionality and precise control of the geometric parameters of drug carrier. Additionally, the high stability of organic polymeric particles in solvents allows the easy loading of hydrophobic drugs from their solutions in alcohol or other solvents into the carriers. In this case, the utilization of highly reproducible and controlled synthetic routes are of the prime importance. In the last decade, an actively developing direction in polymer chemistry has been the synthesis and study of branched polymer systems called polymer brushes (PB). A striking example of such systems are molecular polymer brushes, which are graft copolymers consisting of a backbone of the main chain and side chains covalently attached to it.

In [23,24] methods were developed for the preparation of polymer brushes with polyimide bearing polyvinyl side chains of various natures using controlled ATRP polymerization; a number of high molecular weight multicenter macroinitiators with an adjustable degree of functionalization based on hydroxyl-containing polyimides were synthesized. Using ATRP polymerization of a number of vinyl monomers (methyl methacrylate, *tert*-butyl methacrylate, styrene, *n*-butylacrylate, *tert*-butylacrylate) on multicenter polyimide macroinitiators, new graft copolyimides with homopolymer chains and block copolymers were synthesized. The resulting polyimide brushes, soluble in alcohol and water, with polymethacrylic acid side chains acting as nanocontainers for porphyrazine agents for photodynamic therapy of cancer were applied.

In a previous work it was demonstrated that porphyrazine (Pz) can effectively be adsorbed by the molecular brushes consisting of a hydrophobic polyimide main chain and hydrophilic PMAA side chains [23]. This complex acts as an efficient drug carrier, which is able to penetrate and accumulate inside the tumor tissue [16]. A low molecular weight polyimide (*M* = 7.3 kDa) bearing PMAA side chains with *M* = 13 kDa was used in order to ensure the optimal size of nanoparticles. However, the main disadvantage of such graft-copolymers is the lack of biodegradation and high stability of polyimide main chain. This problem can be resolved by the introduction of biodegradable main chains based on natural polymers. Previously, micelles based on cellulose-graft-poly(*N*,*N*-dimethylaminoethyl methacrylate)-graft-poly(ε-caprolactone) have been prepared and via combination of ring-opening polymerization and ATRP [25]. Such dual graft molecular brushes exhibit the ability to act as drug nanocarriers which have the potential for controlled drug release. The another example of amphiphilic cellulose based brushes was described in [26], where ethyl cellulose was modified with mono and dual side chains of poly(2-(2-methoxyethoxy)ethyl methacrylate)-co-oligo(ethylene glycol) methacrylate)) and poly(2-(*N*,*N*-dimethylamino)ethyl methacrylate) via a combination of ATRP and click-chemistry. The self-assembly behavior and temperature–pH responsive properties of the prepared graft-copolymers were investigated in detail. Although, authors did not report studying the drug sorption and release properties of such systems, they are proposed to have potential in the biomedical applications.

In the present work cellulose macromolecules were used as the main chain for preparation of polymer brushes. Despite the fact that cellulose itself is a hydrophilic polymer, modification of it with an excess of brom-isobutyryl leads to the hydrophobization along with formation of macroinitiator. This fact was used in this work to prepare graft copolymers with hydrophobic cellulose-based main chains with a simpler procedure in comparison with the preparation of graft copolymers with dual types of side chains. Finally, to ensure the optimal size of the nanocontainer, partly hydrolyzed microcrystalline cellulose with reduced molecular weight was used as the starting material.

## 2. Materials and Methods

### 2.1. Materials

Microcrystalline cellulose (MCC) with an average particle size of 50 μm was purchased from Acros Organics (Buchs SG, Switzerland). 2-bromo-isobutyroyl bromide (BiBB), ionic liquid 1-butyl-3-methylimidazolium chloride (BMIMCl), solvent *N*,*N*-dimethylformamide (DMF), copper (I) bromide (CuBr), pentamethyldiethylenetriamine (PMDETA), and *tert*-butyl methacrylate (TBMA) as a monomer (98%) were distilled twice in a vacuum before use, and orthophosphoric acid (99%), for acid hydrolysis was purchased from Sigma Aldrich (Saint-Louis, MS, USA). Trifluoroacetic acid, methylene chloride (reagent grade), tetrahydrofuran (THF) (chemically pure), ethyl alcohol, methyl alcohol, and potassium hydroxide was purchased from Vekton (Saint-Petersburg, Russia).

### 2.2. Synthesis of Cellulose-Polymethacrylate Brush

**Scheme 1:**
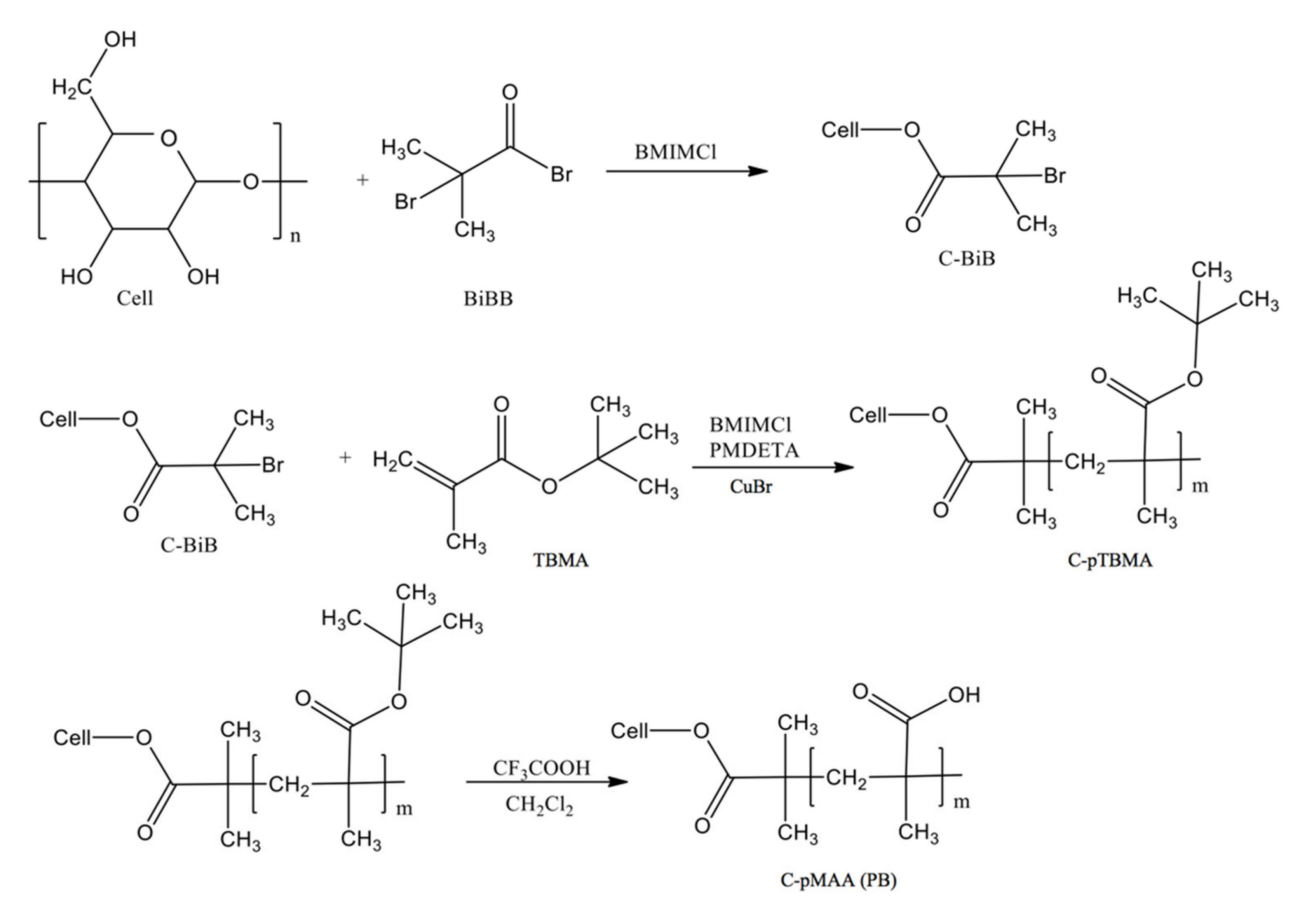
Synthesis of a graft copolymer of cellulose with side chains of poly(methacrylic acid).

Scheme 1 demonstrates the chemical rout for preparation of polymer brush.

#### 2.2.1. Synthesis of Macroinitiator, C-BiB

For this, 0.4 g of MCC was dissolved in 10 g of an ionic liquid–[BMIM]Cl at 80 °C, 5 mL of bromo-anhydride of 2-bromo-isobutyric acid (BiBB) was added dropwise to the solution, the reaction the mixture was kept for 8 h, after which the macroinitiator was planted in an excess of deionized water, filtered, washed, and dried under vacuum to constant weight.

#### 2.2.2. Polymerization of TBMA on a Macroinitiator

For this, 0.035 g of C-BiB was dissolved in 10 g of [BMIM]Cl at 80 °C. The resulting solution was degassed under vacuum, after which 0.01 g of CuBr and 1.5 mL of TBMA was added to it. Before the reaction, TBMA was degassed by threefold repetition of freeze–thaw cycles. The reaction was carried out with stirring on a magnetic stirrer for 7 h and left overnight at room temperature. The resulting product (cellulose-based brush with poly(TBMA) side chains (C-pTBMA)) was planted in an excess of distilled water and purified with acetone on a Soxhlet extraction apparatus.

#### 2.2.3. Molecular Cellulose Brushes with Poly(methacrylic Acid) Side Chains

Freshly distilled trifluoroacetic acid was introduced into a solution of a cellulose-based brush with poly(TBMA) side chains with a concentration of 4 wt% in pre-distilled CH_2_Cl_2_. The reaction was carried out at a temperature of 25 °C with constant stirring for a day, then the solvent was distilled off, the precipitated cellulose-g-poly(methacrylic acid) (C-pMAA) was purified by reprecipitation from ethyl alcohol into methylene chloride, and subsequently dried at 40 °C under vacuum.

### 2.3. Synthesis of Porphyrazines

The synthesis of porphyrazines was carried out by analogy with previous reported studeis [27,28].

### 2.4. Loading of Porphyrazines into Polymer Brushes (PzPB)

**Scheme 2:**
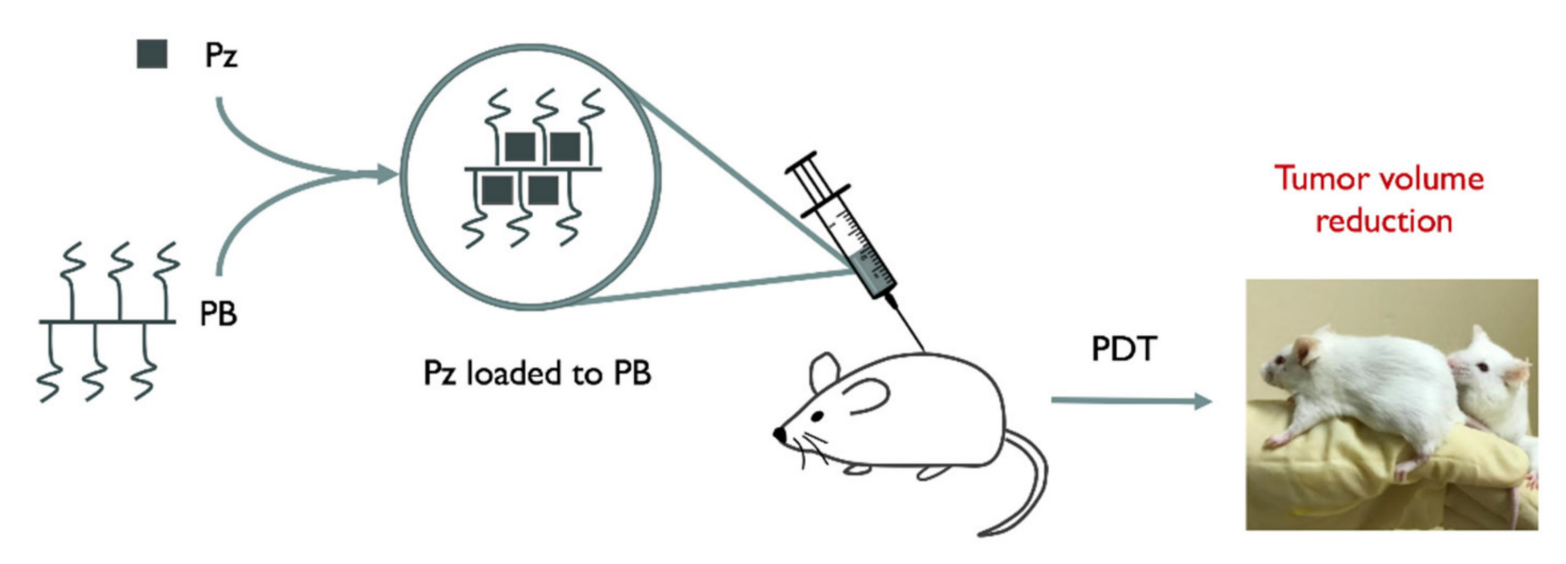
Schematic diagram demonstrating the cancer treatment with cellulose brush loaded with porphyrazine.

A procedure similar to [24] was applied to prepare porphyrazine loaded polymer nanoparticles. Briefly, polymer brushes were loaded with a photosensitizer, Pz (3 mg, 3.1 mmol), that was dissolved in a few drops of ethanol and the resulting solution was diluted with an aqueous solution containing 0.07 wt% PB to obtain a Pz concentration of 1 mM (stock solution). Scheme 2 shows a method for delivering Pz to cancer tumor cells that will be discussed further in Section 2.5.

### 2.5. Biological Experiments with PzPb

#### 2.5.1. Cell Line

Murine colon carcinoma cells CT-26 (ATCC^®^ CRL-2638) were cultured in Dulbecco’s modified Eagle’s medium (DMEM) (PanEco, Moscow, Russia) with 10% (*v*/*v*) fetal calf serum (HyClone, Logan, UT, USA) and 4-mM L-glutamine (PanEco, Moscow, Russia). Cells were grown in 5% CO_2_ at 37 °C. At each stage of passaging, cells were treated with Trypsin–Versene solution (PanEco, Moscow, Russia).

#### 2.5.2. Animals and Tumor Model

Balb/c female mice (7–8 weeks old; 16–20 g) were used. A suspension of 0.5 million CT-26 cells in 50-μL phosphate-buffered saline (PBS, PanEco, Moscow, Russia) was inoculated subcutaneously into the dorsal side of a left hind limb. Experiments were started on day 7 after the cell inoculation, when the tumor volume reached ~0.25 cm^3^. When the size of the tumor node reached 2500 mm^3^, the animals were sacrificed by a cervical dislocation method.

All experimental procedures were approved by the Bioethics Commission of Lobachevsky Nizhny Novgorod State University (Approval No. 07 issued at 4 July 2017 with Supplement No. 39 issued at 31 January 2020).

#### 2.5.3. Analysis of Selectivity of Pz Accumulation in Tumor

Mice were thoroughly shaved with a trimmer, followed by the application of depilatory cream. Pz-PB or Pz solution was injected into the tail vein at a dose of 15 μg of Pz per 1 g of mouse weight (*n* = 3–4).

Whole-body fluorescence imaging was performed using a home-built imaging system [29]. For the procedure, the animals were fixed on a dark low-reflective plate without anesthesia. Fluorescence was excited by LED at a wavelength of 585 nm and collected in the range 645–725. Images were captured before and at different time points after the administration of Pz-PB and Pz and analyzed using ImageJ software (National Institute of Health, Bethesda, MD, USA).

The averaged fluorescence signal was estimated in two regions of interest: tumor area and a region of the same area to the right hind limb (normal tissue). The contrast was defined as the ratio of the two signals.

#### 2.5.4. In Vivo PDT Treatment

Tumor-bearing mice were randomly divided into groups: “control”, “PzPB”, “150 J/cm^2^”, “Pz + 150 J/cm^2^”, and “PzPB + 150 J/cm^2^” (*n* = 3–7). Animals in groups “PzPB”, “Pz + 150 J/cm^2^”, and “PzPB + 150 J/cm^2^” were injected with the corresponding solution as described above. The control mice were administered with PBS.

The tumors of animals in groups “150 J/cm^2^”, “Pz + 150 J/cm^2^”, and “PzPB + 150 J/cm^2^” were irradiated by 640-nm LED light source 3 h post injection at a dose of 150 J/cm^2^ and power density of 100 mW/cm^2^. To prevent undesired tissue heating, the tumor was irradiated in three sessions (50 J/cm^2^, 8.3 min) alternating with 5 min intervals.

The volume of the tumor node was calculated from linear dimensions measured with a caliper. The tumor growth inhibition coefficient (TGI) was calculated using the following equation:

(1) $$TGI\left(\%\right)=\frac{V_{c}-V_{t}}{V_{c}}\times 100\%,$$

where $V_{t}$ and $V_{c}$ are the mean tumor volume in the treated and control groups, respectively. Statistical analysis was performed using one-way ANOVA and Dunnett’s test (GraphPad Prism 6, GraphPad Software, La Jolla California, USA, 2012).

## 3. Results and Discussion

### 3.1. Brush Synthesis and Characterization of Chemical Structure

**Figure 1:**
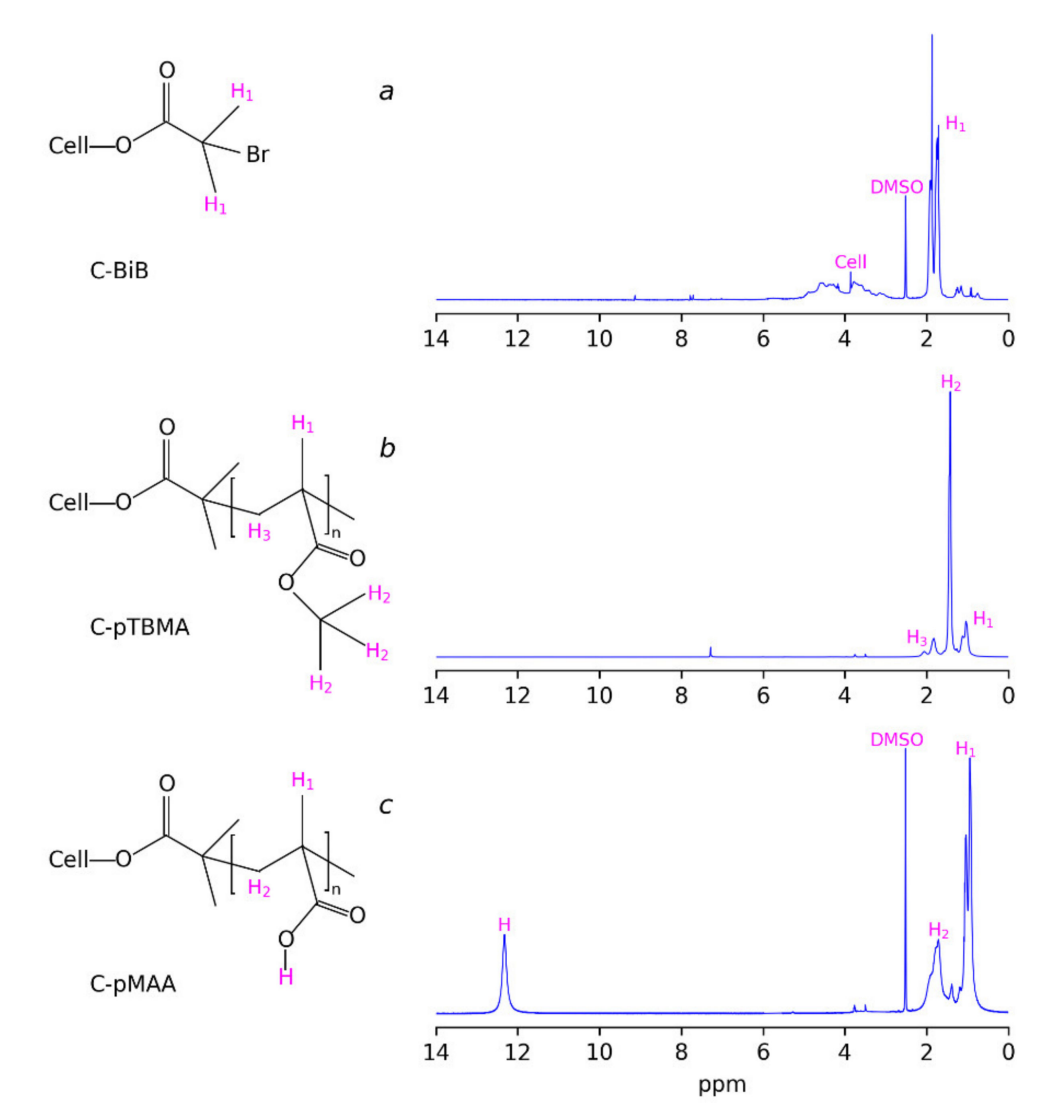
(**a**) ^1^H NMR spectra of macroinitiator C-BiB; (**b**) cellulose-g-poly(*tert*-butylmethacrylate) C-ptBMA, and (**c**) cellulose-g-poly(methacrylic acid) (C-pMAA).

**Figure 2:**
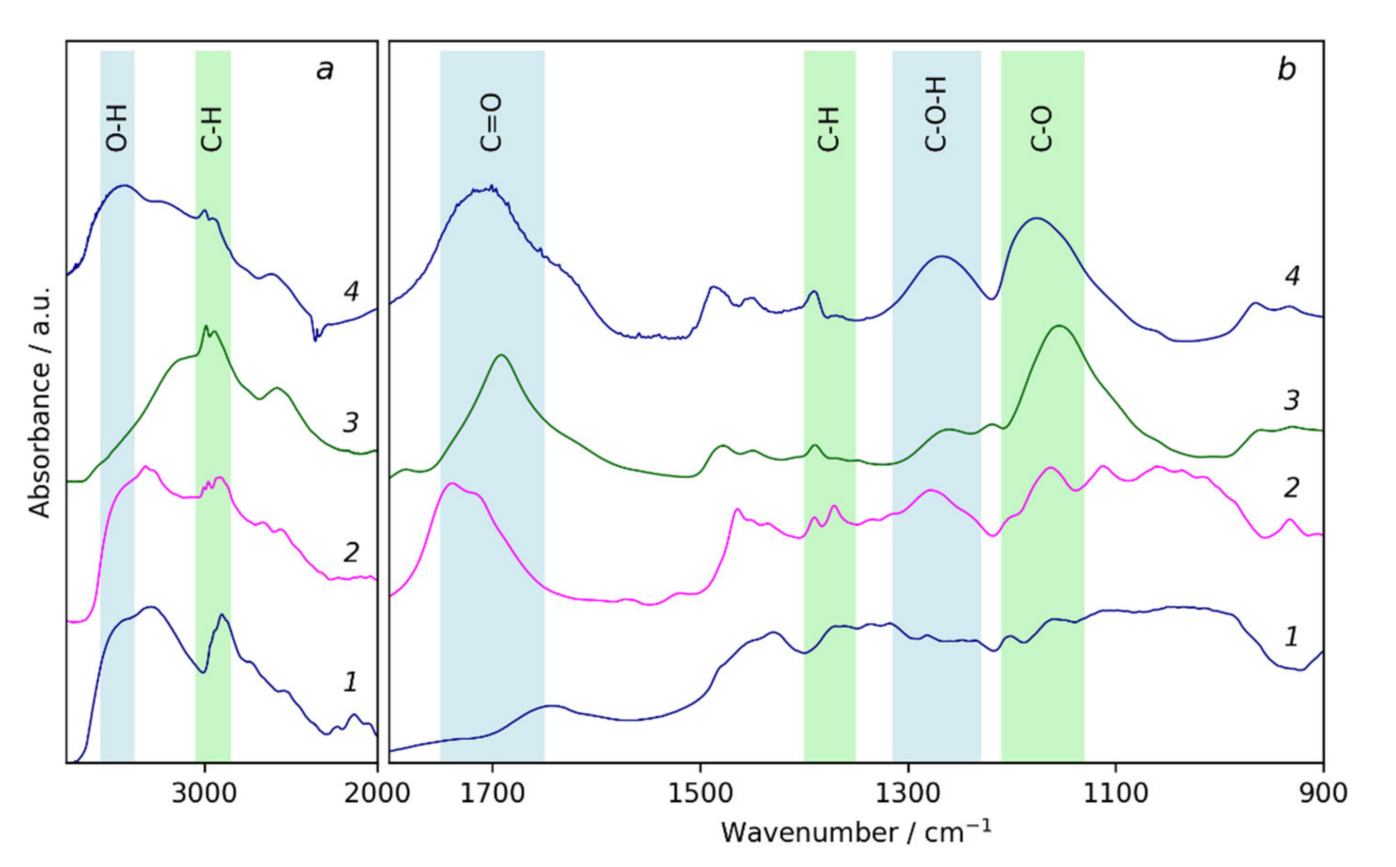
FTIR spectra region 2000–3700cm^−1^ (**a**) and region 900–1900cm^−1^ (**b**) of initial microcrystalline cellulose (1), macroinitiator C-BiB (2), cellulose-g-poly(*tert-*butylmethacrylate) C-pTBMA (3), and cellulose-g-poly(methacrylic acid) (4).

The macroinitiator (C-BiB) was obtained by the reaction of MCC dissolved in [BMIM]Cl with 2-bromo-isobutyric acid bromide. The successful addition of bromine anhydride to the cellulose chain is proved with the ^1^H NMR spectra (Figure 1a). The spectra demonstrate the characteristic peaks of the hydrogens of –CH_3_ groups of isobutyryl bromide at 1.5–2 ppm and two broad peaks, which can be attributed to the hydrogens in cellulose. The absence of a signal of hydrogens in carboxyl groups in the ^1^H NMR spectrum in the region of 11–12 ppm can be noticed. This can be considered as the indicator of the absence of 2-bromo-isobutyric acid resulting from possible hydrolysis during precipitation of the reaction mass in water. Thus, there was no free brom-isobutyryl bromide in the product. Additional evidence for the successful formation of macroinitiator is given by the FTIR spectra of initial MCC and C-BiB, which are given in Figure 2, curves 1 and 2, respectively. The spectra of C-BiB in comparison with initial MCC demonstrate appearance of strong band at 1740 cm^−1^, which is typical for C=O groups. The doublet at 1370 and 1390 cm^−1^ is characteristic of isopropyl group in BIBB; bands near 930 cm^−1^ can be attributed to the rocking vibrations of –CH_3_ groups. Along with the appearance of band characteristic of methyl groups at 2990 cm^−1^, these observations prove the formation of the macroinitiator from the initial cellulose macromolecules. Finally, based on the ratio between ^1^H NMR signals of the BiB in the range 1.51–2.1 ppm (see Figure 1) and cellulose signals in the range of 2.74–6.33 ppm, the number of attached BiB molecules to cellulose was estimated as two per one cellulose unit.

At the next stage, the ATRP was performed using *tert*-butyl methacrylate as the monomer. The typical ^1^H NMR spectra of the polymerization product (C-pTBMA) is given in Figure 1b where peaks at 1.04 and 1.85 ppm, corresponding to the hydrogens of –CH_3_ and –CH_2_– groups of methacrylic acid, respectively, are seen. The intense peak at 1.42 ppm corresponds to the –CH_3_ groups of *tert-*butyl- radicals. FTIR spectra of C-pTBMA (Figure 2, curve 3) demonstrate significant decreasing of the intensity of the –OH absorption band at 3400–3600 cm^−1^, which is a result of the decreasing of the mass fraction of cellulose in the sample due to the formation of the polymer. At the same time, the intensity of a doublet with maximums at 2993 and 2936 cm^−1^ increased significantly, which demonstrates the incorporation of *tert*-butyl groups. In the region 800–1700 cm^−1^ the spectrum of C-BiB is completely masked with peaks, corresponding to poly(*tert*-butylmethacrylate). The most intensive absorption bands of C-pTBMA are centered at 1691 and 1154 cm^−1^, which correspond to the vibrations of C=O and C–O bounds in the ester group, respectively. Thus, spectroscopic data confirms the formation of poly(*tert*-butylmethacrylate) side chains on C-BiB macroinitiator.

Molecular characteristics of the brushes were changed by variation of the ratio of the macroinitiator and *tert*-butyl methacrylate. In order to calculate the average length of side chains, C-pTBMA samples were characterized by gel permeation chromatography: the number average molecular weights were 6.8, 180, 370, and 500 kDa for the macroinitiator and brushes synthesized at BiBB to monomer ratios of 1:50, 1: 100, and 1: 200, respectively. Assuming the equivalence of all polymerization centers on the macroinitiator, it is possible to estimate the average length of poly(*tert*-butylmethacrylate) chains grafted to cellulose as 40, 85, and 116 monomorphic units for brushes C-pMAA-1, C-pMAA-2, and C-pMAA-3, respectively.

At the final stage, the resulting C-pTBMA graft copolymer was subjected to hydrolysis in CH_2_Cl_2_ with CF_3_COOH, which resulted in the removal of *tert*-butyl groups and the formation of the final product: C-pMAA (PB). The completeness of the hydrolysis during the removal of the *tert*-butyl groups was monitored using ^1^H NMR spectroscopy (Figure 1c). It was seen that, the peak at 1.42 ppm, corresponding to the *tert*-butyl radicals disappeared. At the same time, an additional peak at 12.28 ppm appeared, which can be attributed to the formation of carboxylic acid. On the FTIR spectra (Figure 2 curve 4) the appearance of broad band at 3300–3600 cm^−1^ and intensive peak at 1267 cm^−1^ corresponding to the vibration of O–H and C–OH bounds, respectively, were observed, which confirms the successful formation of cellulose-g-poly(methacrylic acid) brushes. The obtained macromolecules are soluble in water and in alcohols, which is significant for their using as containers for sorption and delivery of porphyrazine photosensitizers, as it will be discussed further.

### 3.2. Drug Sorption in Brushes

**Figure 3:**
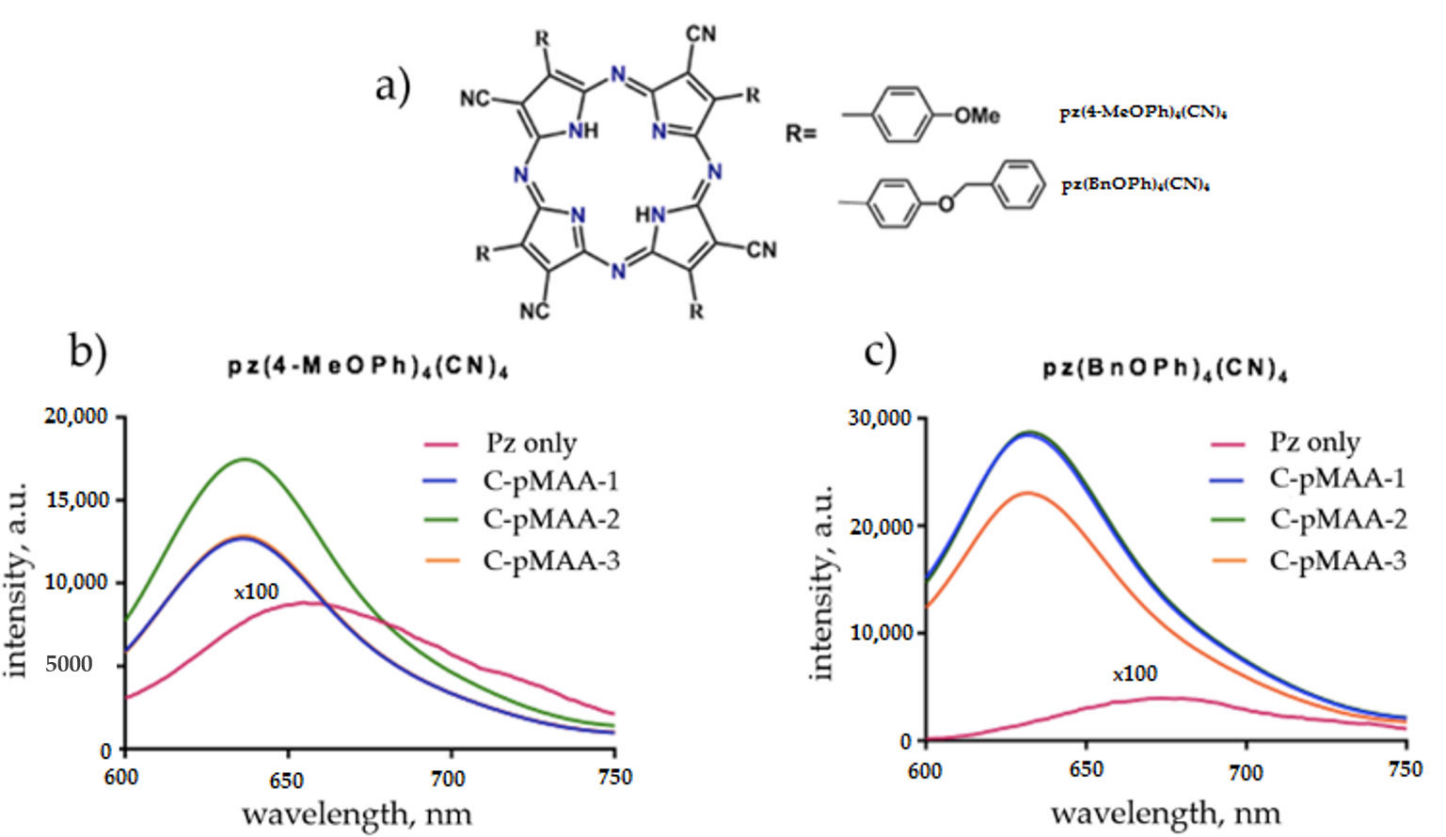
(**a**) The chemical structure of porphyrazines and the fluorescence spectra of solutions of porphyrazines (**b**) (pz(4-MeOPh)_4_(CN)_4_) and (**c**) (pz(4-BnOPh)_4_(CN)_4_) in the free state and in the presence of C-pMAK of various molecular structure. The excitation wavelength is 585 nm.

The obtained C-pMAA samples, unlike the initial cellulose and its copolymers with poly(*tert*-butyl methacrylate), are soluble in water and are of interest as nanocontainers capable of sorbing and delivering drugs in vivo. To test the sorption properties of the graft copolymers obtained in this work, the effect of the molecular structure of modified cellulose on its ability to bind porphyrazine photosensitizers, was studied using fluorescence spectroscopy. For this purpose, types of porphyrazins containing 4-methoxy-phenyl (pz(4-MeOPh)_4_(CN)_4_) and 4-benzoxy-phenyl (pz(4-BnOPh)_4_(CN)_4_) substituents (Figure 3a) were used. The fluorescence spectra of the solutions of which in free form and in the presence of C-pMAA are shown in Figure 3b and Figure 3c, respectively.

It turned out that for all samples of modified cellulose, a significant increase in the fluorescence of porphyrazines was observed, indicating the effective binding of the drug to the molecules of modified cellulose (Figure 3b,c). Moreover, in the case of (pz(4-MeOPh)_4_(CN)_4_), the most effective binding agent was C-pMAA-2, while for (pz(4-BnOPh)_4_(CN)_4_), C-pMAA-2 and C-pMAA-3 were effective.

### 3.3. Anticancer Activity Testing

#### 3.3.1. Accumulation of Drug in Tumor Tissue

The sensitizers for photodynamic therapy (PDT) based on cyano-aryl porphyrazine pigments ensure high survival of experimental animals that have undergone a therapeutic procedure. For biological experiments in this study, a C-pMAA-2 polymer brush was chosen as nanocontainer, which was loaded with pz(4-MeOPh)_4_(CN)_4_ photosensitizer. In the subsequent text the abbreviation PzPB will denote this combination of brush and drug.

**Figure 4:**
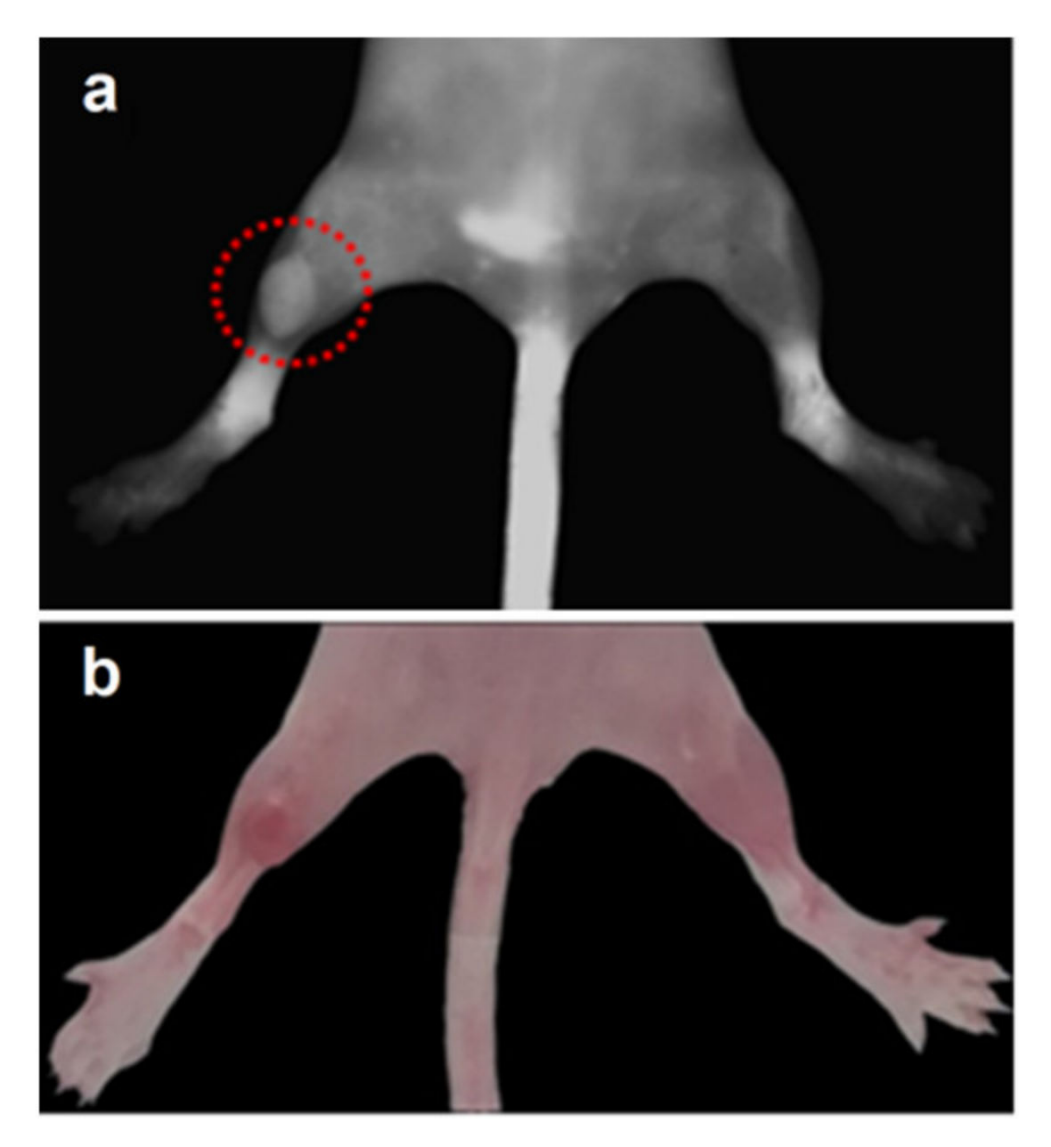
Fluorescent image (**a**) and photo (**b**) of a tumor-bearing mouse 2 h after intravenous administration of PzPB (Pz concentration of 15 mg/kg). Fluorescence was registered at *λ_ex_* = 585 nm, *λ_em_* = 645–725 nm. The dotted red circle marks the tumor area.

**Figure 5:**
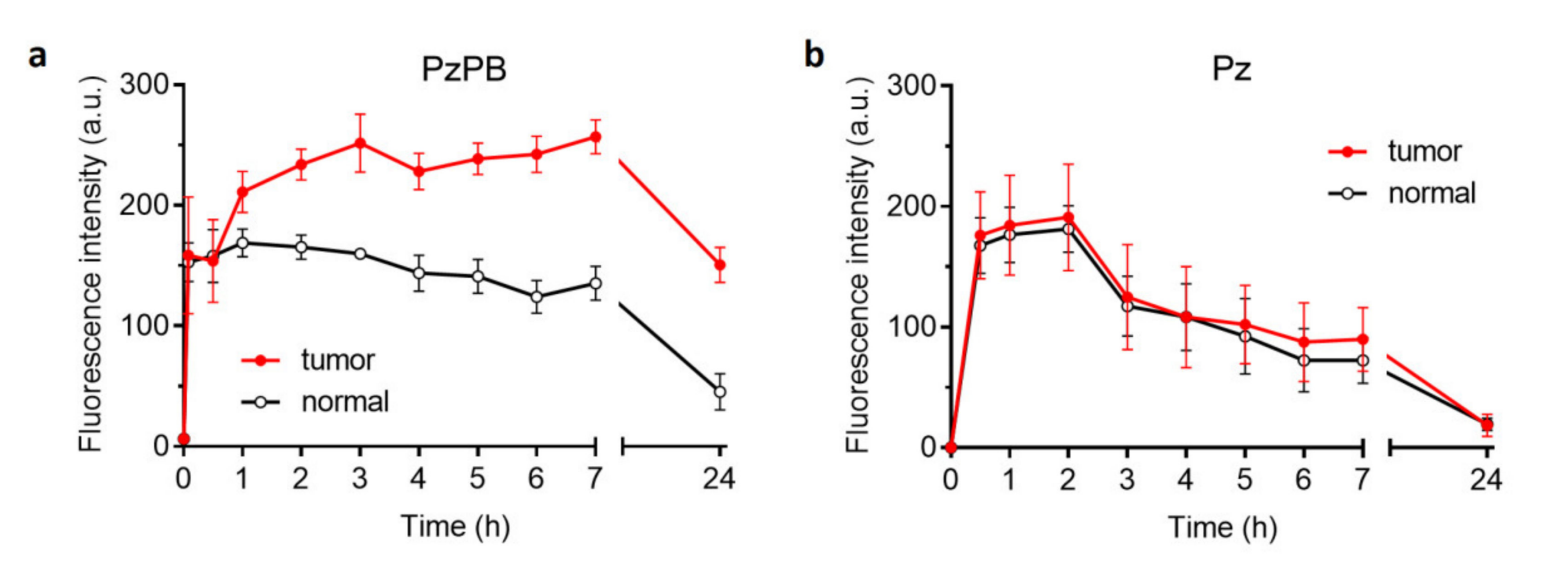
Dynamics of changes in the fluorescent signal in the tumor (left hind paw of the mouse) and muscle tissue (right hind paw of the mouse) according to the data of fluorescence imaging after intravenous administration of PzPB (**a**) or Pz (**b**). Pz concentration is 15 mg/kg in both cases. Error bars are represented by the standard deviation.

The fluorescent image of a tumor-bearing mouse given in Figure 4a demonstrates that the fluorescent intensity of the tumor is obviously higher than for surrounding tissues after 2 h of intravenous administration of PzPB loaded in polymer brush. PzPB provided selective accumulation of Pz in the tumor and retention for 7 h after administration (Figure 5a). In the case of porphyrazine administration without a carrier, the ratio of signals in the tumor and in the norm is close to unity (Figure 5b). This demonstrates the effective EPR effect of the synthesized polymer brushes and their ability to penetrate inside tumor tissue via the capillary network. The data on dynamic changes of contrast of the fluorescent signal between the tumor (left hind paw of the mouse) and muscle tissue (the right hind paw of the mouse) according to the data of fluorescence imaging after intravenous administration of PzPB are given in Table 1. It can be seen that the contrast increased gradually from 1.3 after 1 h up to 3.3 after 24 h, thus demonstrating continued accumulation of the drug into tumor tissue during a prolonged period of time.

#### 3.3.2. Photodynamic Treatment with PzPB

**Figure 6:**
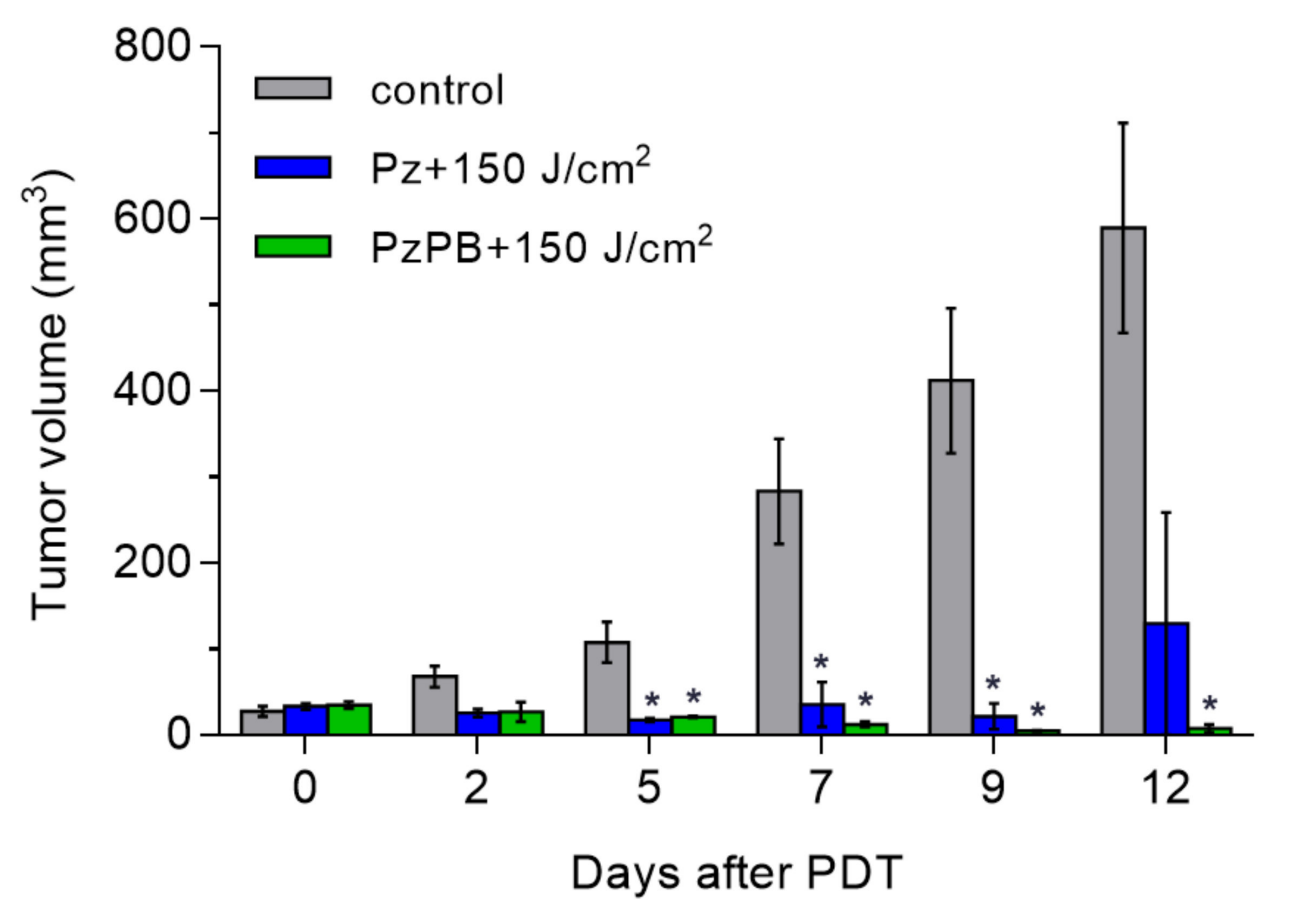
Dynamics of changes in the volume of the tumor node in the control and experimental groups after PDT with PzPB or Pz. Error bars are represented by the standard error of the mean. *, statistically significant difference from the control group, ANOVA, *p* < 0.05.

At the next stage, the irradiation of animals after PzPB accumulation in tumors at a dose of 150 J/cm^2^ was applied. This lead to the regression of the tumor as can be seen in Figure 6, showing the dependence of the volume of the tumor on time. In vivo exposure of CT-26 tumors to tetra(4-fluorophenyl)tetracyanoporphyrazine loaded into polymer brushes resulted in complete recovery for five out of seven experimental animals. The effect of treatment with Pz without carrier resulted in relapse of rapid tumor growth in 12 days. Higher therapeutic effect for PzPB presumably resulted from higher Pz concentrations reached in tumors at the 3 h time point when the irradiation of the tumors was performed.

**Figure 7:**
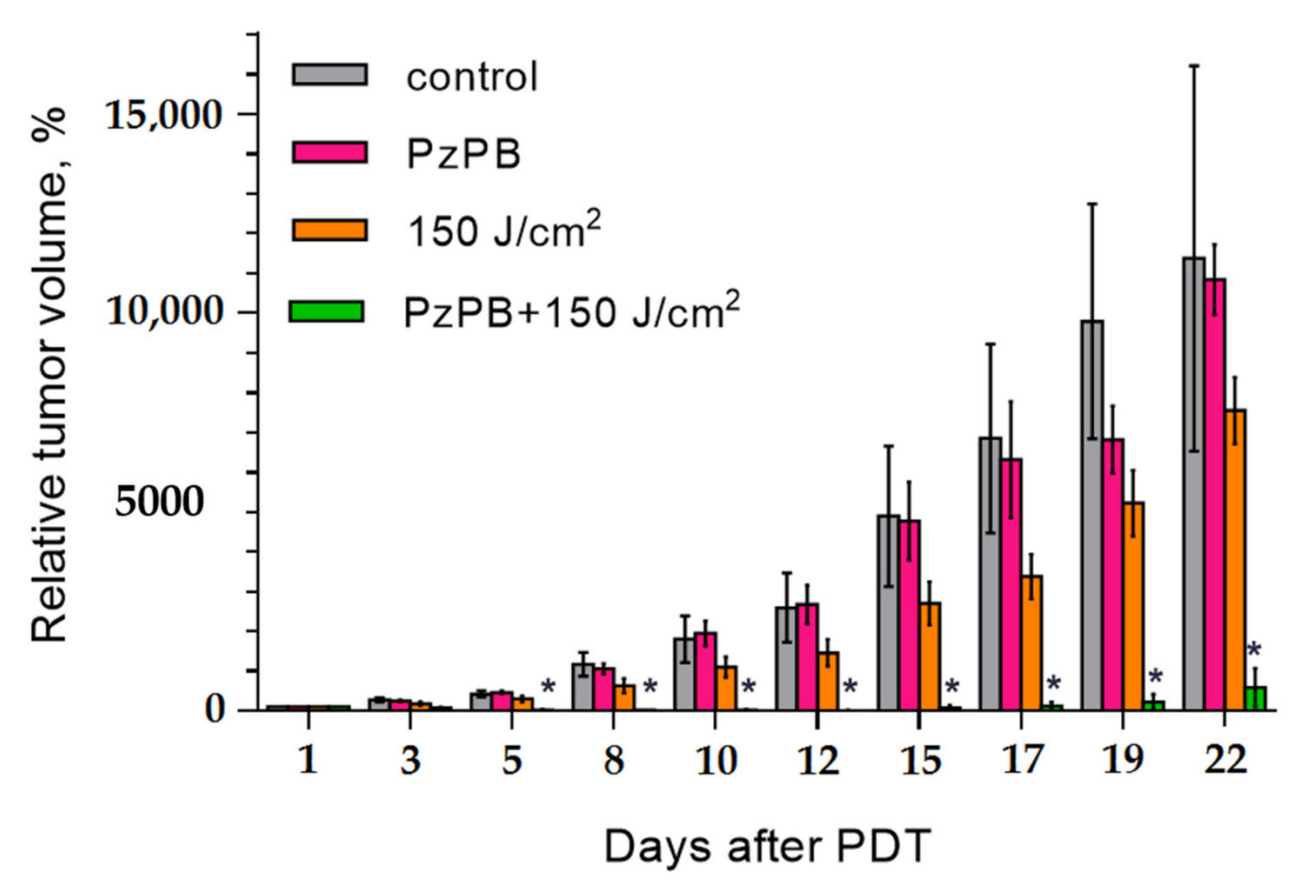
Time dependence of relative tumor volume after PDT with PzPB. Error bars are represented by the standard error of the mean; *- statistically significant difference from the control group, ANOVA, *p* < 0.05.

**Figure 8:**
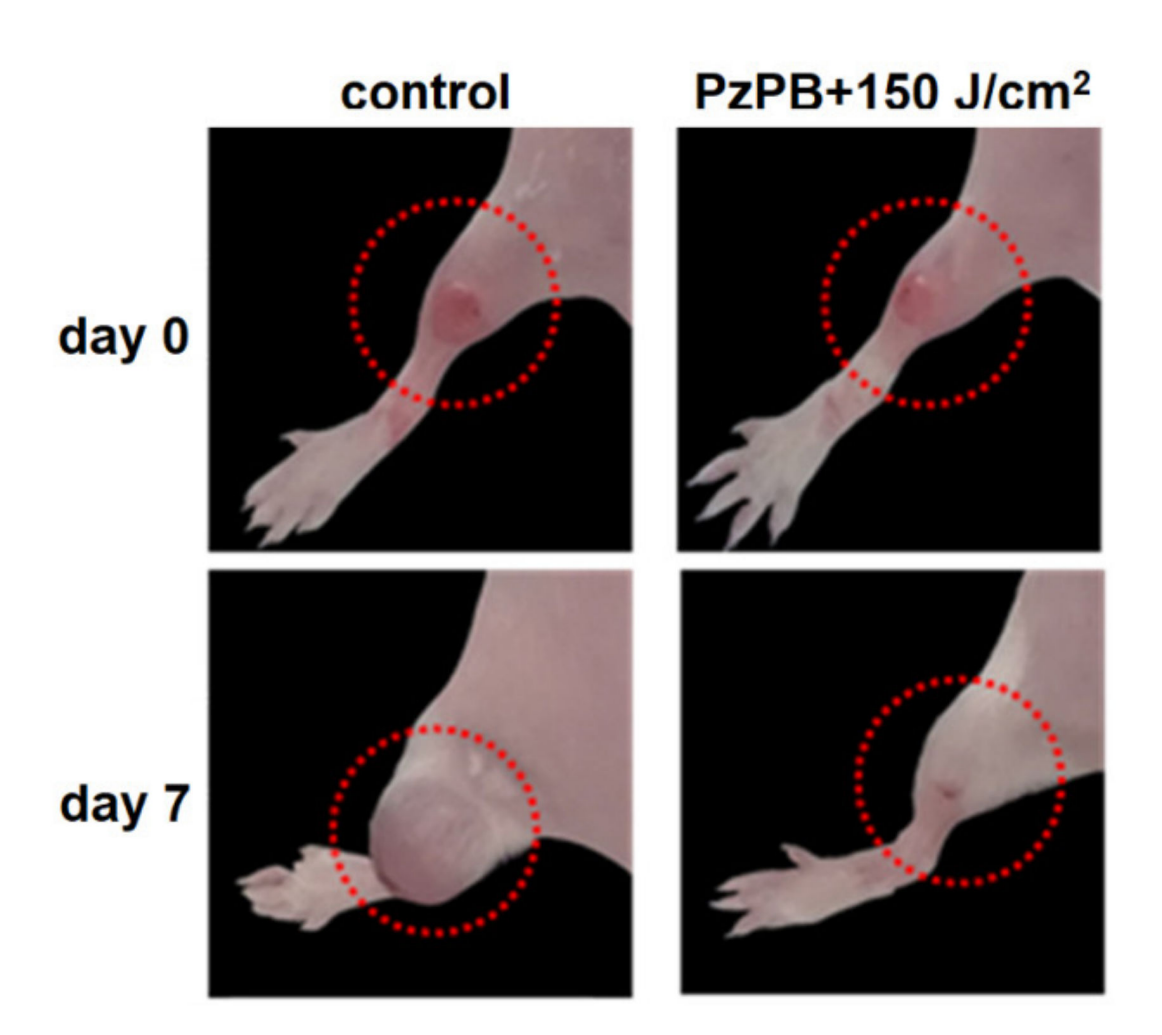
Hind paws of the mice with experimental tumors in control group and group treated with PzPB-based PDT. The images were obtained before and 7 days after treatment. The dotted red circle marks the tumor area.

In order to verify the effect of the combination of PDT with PzPB the experiments were carried also in which only irradiation or administration of PzPB was applied. The results are given in Figure 7. It can be seen that the therapeutic effect occurs only for the combination of administration of PzPB and irradiation. The photos given in Figure 8 illustrate the reduction of tumor volume after 7 days for mice subjected the PDT with PzPB in comparison with animals from control group. To compare the effects on various treatment options on tumor growth, we calculated the tumor growth inhibition (TGI) coefficient. For the PzPB-PDT group, TGI was 99% at 12 day, and about 95% at 22 day after treatment. We should note the moderate effect of irradiation itself, with a TGI of about 30% at 22 day, however, the effect was not statistically significant.

## 4. Conclusions

ATRP was used to obtain water soluble cellulose-g-poly(methacrylic acid) brushes. The ability of the prepared nanoparticles to perform the role of nanocontainers for carrying porphyrazine-type drugs was demonstrated. The carrier was successfully loaded with new types of porphyrazines containing 4-methoxy-phenyl (Pz(4-MeOPh)_4_(CN)_4_) and 4-benzoxy-phenyl(Pz(4-BnOPh)_4_(CN)_4_), as was demonstrated with fluorescent spectroscopy. After the intravenous introduction of the cellulose brushes into the bodies of mice, the selective accumulation of the drug in tumors was observed with fluorescent imaging that demonstrated ability of the prepared polymer brushes to ensure the permeability and retention effect. It was demonstrated that the prepared nanocontainers loaded with porphyrazines can act as an effective reducing agent of tumors during photodynamic therapy.

## Figures and Tables

**Table 1:** Dynamics of tumor-to-normal tissue contrast of the fluorescent signal measured by whole-body fluorescence imaging after intravenous administration of PzPB (Pz concentration 15 mg/kg).

Time (h)	Contrast
1	1.3
2	1.4
3	1.6
4	1.6
5	1.7
6	2.0
7	1.9
24	3.3

## Data Availability

Data presented in this article is available on request from the corresponding author.

## Institutional Reviewer Board Statement

The study was conducted according to the guidelines of the Declaration of Helsinki and approved by the Bioethics Commission of Lobachevsky Nizhny Novgorod State University (Approval No. 07 issued at 4 July 2017 with Supplement No. 39 issued at 31 January 2020).
